# Effect of Core Exercises on Motor Function Recovery in Stroke Survivors with Very Severe Motor Impairment

**DOI:** 10.3390/jcdd10020050

**Published:** 2023-01-28

**Authors:** Zuliana Bacho, Nyein Yin Khin, D Maryama Ag. Daud

**Affiliations:** 1 Sports Science Program, Faculty of Psychology and Education, University Malaysia Sabah, Kota Kinabalu 88400, Malaysia; 2 Department of Rehabilitation Medicine, Faculty of Medicine and Health, University Malaysia Sabah, Kota Kinabalu 88400, Malaysia; 3 HEAL Research Unit, Faculty of Medicine and Health, University Malaysia Sabah, Kota Kinabalu 88400, Malaysia; 4 Department of Biomedical Sciences, Faculty of Medicine and Health, University Malaysia Sabah, Kota Kinabalu 88400, Malaysia

**Keywords:** stroke, motor impairment, motor function, core exercises

## Abstract

Paresis of the upper and lower limbs is a typical issue in stroke survivors. This study aims to determine whether core exercises help stroke survivors with very severe motor impairment recover their motor function. This study employed a within-subjects design. Eleven hemiparetic stroke patients with very severe motor impairment (FMA score < 35) and ages ranging from 24 to 52 years old were enrolled in this study. All participants engaged in supervised core exercise training twice a week for 12 weeks. The main outcome measures were Fugl-Meyer Assessment Lower Extremity (FMA-LE) and Fugl-Meyer Assessment Upper Extremity (FMA-UE), which were measured before training and at intervals of four weeks during training. Repeated measures ANOVA was used to analyze the effect of core exercises on motor function performance and lower extremity motor function and upper extremity motor function recovery. There were significant differences in the mean scores for motor function performance, lower extremity motor function, and upper extremity motor function throughout the four time points. A post-hoc pairwise comparison using the Bonferroni correction revealed that mean scores significantly increased and were statistically different between the initial assessment and follow-up assessments four, eight, and twelve weeks later. This study suggests that 12 weeks of core exercise training is effective for improving motor function recovery in patients with very severe motor impairment.

## 1. Introduction

Motor function impairment is the most frequent impairment diagnosed in stroke and is defined as a loss or limitation of function in muscle control or movement or limitation of movement [1]. Impaired upper limb function causes patients difficulty when performing daily tasks such as moving and coordinating the arms, hands, and fingers on the paretic side or during reaching tasks. While lower limb impairment will affect the patient’s gait, transfer, and mobility [2].

The development of movement control and stability occurs in a core-to-extremity (proximal-to-distal) progression [3]. During whole-body movements, transversus abdominis, multifidus, rectus abdominis, and oblique abdominal muscles are consistently recruited before any limb movement [4]. In this situation, the core muscles provide dynamic stability by stabilizing the spine, pelvis, and shoulder girdle, and also provide a strong foundation for limb movement.

Based on a meta-analytical study by Alhwoaimel et al. [5], there are only 17 studies (involving 599 stroke patients) related to core training or trunk exercises and their impact on trunk performance and/or upper limb function post stroke. None of the studies reported the effect of core training (trunk exercises) on functional movement or upper limb impairments. Furthermore, inconsistent results were reported for core performance and gait [6,7,8,9], while no evidence to support the effectiveness of core exercise on upper limb function was provided [2,5]. Therefore, this study was carried out to determine the effects of core exercise on motor function performance in stroke survivors with very severe motor impairments.

## 2. Materials and Methods

### 2.1. Study Design

A repeated measures design with multiple post-tests was employed in this study to determine the effects of core exercise on motor function performance in stroke survivors. Prior to the experimental exercise training session, participants visited the laboratory to undergo health screening and preliminary measurements (i.e., motor function performance, lower extremity motor function, upper extremity motor function, and resting blood pressure). Exercise sessions were conducted in a temperature-controlled laboratory between 20 and 24 °C and exercise sessions were separated by at least 2 days. During the assessment week, participants were instructed not to engage in any exercise session. Participants then performed motor function performance assessments.

### 2.2. Study Population

In this study, the sample was ischemic and hemorrhagic stroke patients who were at least 3 months removed from stroke. Current AHA (2017) [10] statistics states that nearly 10 percent of all strokes occur in individuals 18 to 50 years of age. In Malaysia, there was a substantial increase in stroke incidence in those under 65 years of age [11,12]. Therefore, a pool of 260 stroke patients aged between 20 and 60 years old (particularly those from Kota Kinabalu and the surrounding districts, including Penampang, Tuaran, Putatan, and Papar) who had received outpatient treatment at the Department of Rehabilitation Medicine at Queen Elizabeth I Hospital or Tuaran Hospital (Sabah, Malaysia) were recruited.

The following inclusion criteria were applied: (i) unilateral hemiplegia or hemiparesis; (ii) at least three months post stroke; (iii) medically stable (not confined to bed rest and no restricted mobility due to medical reasons); (iv) normal cognitive level as indicated by a minimum score of 24 out of 30 in the Mini-Mental State Examination (MMSE); (v) a Fugl-Meyer Assessment (FMA) score <35, which denotes very severe motor impairment; and (vi) ability/availability to come to the research centre twice a week.

**Figure 1:**
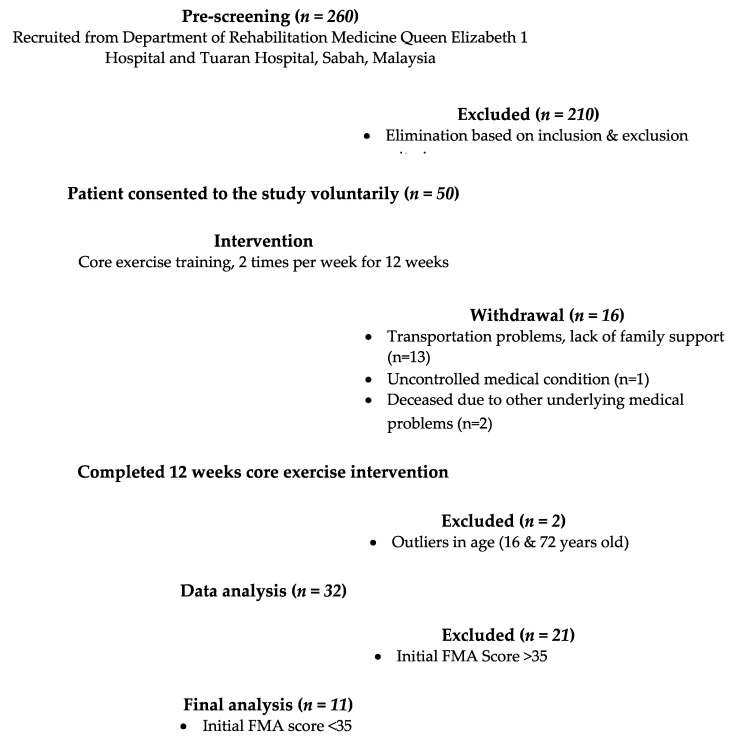
Flow chart of patient recruitment.

Stroke diagnosis was made based on their medical history and clinical evaluations by certified medical doctors. Out of 260 patients, only 50 met the inclusion criteria, with 210 patients thus excluded (Figure 1). Participants with unstable medical conditions were excluded (severe respiratory and cardiac diseases, uncontrolled hypertension and diabetes, and mental illness).

The most frequent reasons for exclusion were the inability to come to the research centre twice a week due to transportation problems and a lack of family and financial support. All 50 patients had given their consent to participate in this study, but only a total of 34 patients (*n* = 34) were able to complete the 12 weeks of comprehensive core exercise intervention. The reasons for discontinuation include transportation problems, a lack of family support, and uncontrolled medical conditions (two patients passed away due to other underlying medical problems). However, 2 patients were excluded from the analysis due to being outliers in terms of age (16 and 73 years old). Therefore, the total number of subjects left was 32 patients. Out of 32, only 11 patients were classified with very severe motor impairment (FMA score less than 35).

### 2.3. Core Exercise Training Intervention

After screening for the inclusion and exclusion criteria, 11 patients with FMA scores less than 35 completed the 24 core exercise training sessions (2 sessions per week). The core exercise training employed in this study consisted of four levels of progression, namely supine exercise, sitting exercise, core strengthening exercise, and dynamic core stabilization as previously prescribed [13]. Every participant was prescribed a 60 min exercise session with the progression increased in stages according to Table 1.

Each training session was closely supervised to ensure all participants performed the exercises with proper technique and obtained an appropriate amount of exercise and rest intervals. The exercise trainer offered further verbal instructions and manipulative inductions, and also assisted the movement if needed. Blood pressure and heart rate were monitored regularly in every session. Furthermore, to minimize discomfort and injury, each exercise session started and ended with stretching.

The study protocol was screened and ethical approval was obtained from the Medical Research and Ethics Committee, Ministry of Health Malaysia, NMRR-16-38-28777 (IIR). Permission to approach the stroke patients and access their case notes was given by the director of Queen Elizabeth I Hospital.

### 2.4. Outcome Measures

#### 2.4.1. Fugl-Meyer Assessment

The FMA scale is divided into four domains: motor functioning (upper and lower extremities), sensory functioning, joint range of motion, and joint discomfort. The motor domain contains questions that evaluate movement, coordination/speed, and reflex responses of the upper extremity’s shoulder, elbow, forearm, wrist, and hand, as well as the lower extremity’s hip, knee, and ankle [14]. Each item consists of movements reflecting motor function in post-stroke hemiparesis, and these movements range from the proximal to the distal joints.

#### 2.4.2. Clinically Significant Motor Function Performance, Lower Motor Function Performance, and Upper Motor Function Performance

Although statistical significance (*p* < 0.05) has been utilized to assess the outcomes of this study, it only provides a limited amount of information to clinicians. Therefore, evaluating clinical relevance can make it easier to translate the research findings from this study to clinical practice. To determine the clinical relevance of this study, the researchers used these criteria for scoring [15]:

i. If effect size (ES) and the mean difference between groups are higher than both MIDs (minimal important differences), it is scored as clinically relevant (CR).
ii. If ES is moderate and one of the MIDs is accomplished, it is scored as potentially clinically relevant (PCR).
iii. If ES is small–moderate and one of the MIDs is accomplished, it is scored as potentially clinically relevant (PCR).
iv. If ES is small and one of the MIDs is accomplished, it is scored as not clinically relevant (NCR).
v. If both ES and MIDs are not accomplished, then it is scored as NCR.

The magnitude of effect size has previously been interpreted as an index of clinical significance [16,17]. The larger this effect size index, the greater the difference between groups or between pre- and post-intervention outcomes, and the greater the clinical relevance of the findings [17]. According to Cohen [18], effect sizes of 0.2, 0.5, and 0.8 were categorized as small, moderate, and large, respectively. Decisions regarding the extent of the effects are made using these values as a guide [19,20]. However, an effect size of > 0.4 was considered clinically relevant since this effect or difference could represent a moderate effect that might be of interest to clinical practice [18]. Mean MID has been defined as “the smallest difference in score in the domain of interest that patients perceive as important, either beneficial or harmful, and which would lead the clinician to consider a change in the patient’s management” [21]. For interpretation purposes, a mean difference between groups that is higher than the MID can be considered clinically relevant [17,22].

### 2.5. Data Analysis

Repeated measures ANOVA was used to analyze the effect of core exercises on motor function performance and lower extremity motor function and upper extremity motor function recovery using Statistical Package for Social Science (SPSS) version 20.0. The level of significance was set at *p* < 0.05. If a significant effect was found, further post-hoc pairwise comparison with the Bonferroni correction was utilized to explore the significant changes across time points.

## 3. Results

### 3.1. Characteristics of Patients

Table 2 summarizes the characteristics of all patients. All 11 patients unilaterally presented hemiparesis and demonstrated normal cognitive function. There were six male and five female patients, with a mean age of 41.0 ± 10.00 years. Patients were grouped into four age groups: 20–29 years (2, 18.2%), 30–39 years (2, 18.2%), 40–49 years (5, 45.5%), and 50–59 years (2, 18.2%). The mean elapsed time since stroke occurrence at the initiation of the intervention was 12 ± 7 months. A total of six patients (54.5%) had had a hemorrhagic stroke and five patients (45.5%) had had an ischemic stroke. Six patients (54.5%) suffered from right hemiparesis, while five patients (45.5%) presented left hemiparesis.

### 3.2. Duration of Stroke

As presented in Table 3, the mean time since onset of stroke at the initiation of the intervention was 12 months, with a mean duration of 12 months for men and 10 months for women. The 50 to 59 age group had the longest mean length (14 months), followed by the 40 to 49, 20 to 29, and 30 to 39 age groups with means of 12, 11, and 8 months, respectively.

### 3.3. Motor Function Performance (MFP) Changes across Time

**Figure 2:**
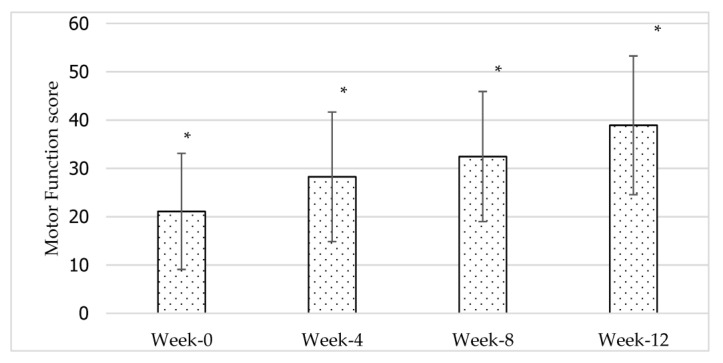
The changes in motor function performance across the 12-week core exercise intervention. *n* = 11; *: significant at *p* < 0.05.

Repeated measures ANOVA analysis (Table 4) showed that there were significant differences in mean MFP throughout the four time points: *F*(1.616, 14.545) = 57.164, *p* < 0.01, *ηₚ*^2^ = 0.86. Given that the (partial eta squared, *ηₚ*^2^) measure of effect size was 0.86, this indicates that 86% of the total variance (main effect and error) was associated with the time points. It is thus likely that time points have a large effect on motor function performance scores. A post-hoc pairwise comparison using the Bonferroni correction revealed that all the time point pairs were statistically significant *p* < 0.01 (refer to Figure 2). The results also demonstrate that there was no interaction effect between time and gender [*F*(1.62) = 0.726, *p* > 0.05] or between time and stroke type [*F*(1.46) = 0.398, *p* > 0.05] for MFP.

### 3.4. Lower Extremity Motor Function (LEMF) Changes across Time

**Figure 3:**
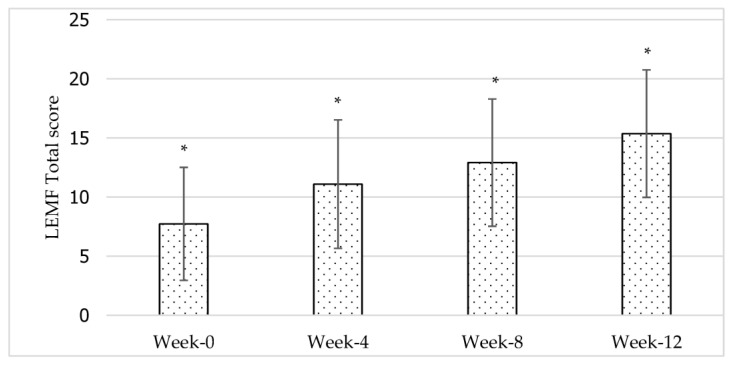
The changes in lower extremity motor function across the 12-week core exercise intervention. *n* = 11; *: significant at *p* < 0.05.

The repeated measures ANOVA findings (Table 4) determine that the mean for LEMF statistically differed significantly between time points (*F*(1.676, 34.139) = 32.509, *p* < 0.01, *ηₚ*^2^ = 0.78) across the 12-week core exercise intervention. The effect size, *ηₚ*^2^, was 0.78, suggesting that time points had a large effect on lower extremity motor function scores. Results of the post-hoc pairwise comparison using the Bonferroni correction (see Figure 3) indicate that all the time points pairs were statistically significant (*p* < 0.01). This means that there is a significant difference between each time point and the lower extremity motor function score. This study also demonstrated that there was no statistically significant interaction effect between time and gender [*F*(1.68) = 0.501, *p* > 0.05] or between time and stroke type [*F*(1.75) = 1.068, *p* > 0.05] for LEMF.

### 3.5. Upper Extremity Motor Function (UEMF) Changes across Time

**Figure 4:**
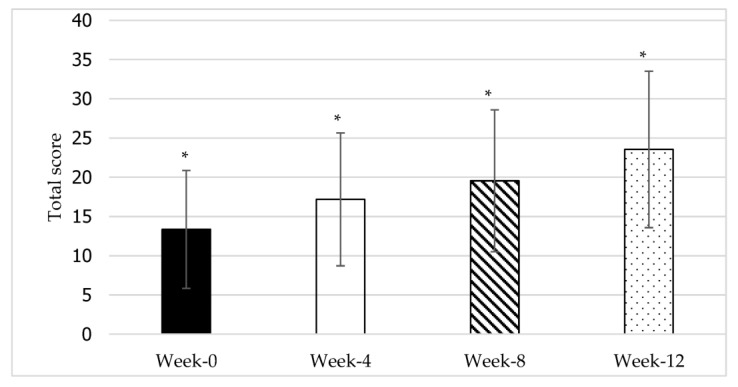
The changes in upper extremity motor function (UEMF) across the 12-week core exercise intervention. *n* = 11; *: significant at *p* < 0.05.

Repeated measures ANOVA (Table 4) demonstrated that time had a statistically significant impact on the recovery of UEMF: *F*(1.416, 12.748) = 28.282, *p* < 0.01, *ηₚ*^2^ = 0.76. The effect size, *ηₚ*^2^, was 0.76, indicating that time points had a large effect on upper extremity motor function scores. Additional post-hoc pairwise comparisons using the Bonferroni correction (Figure 4) revealed that all the time point pairs were statistically significant (*p* < 0.01). This indicates that the upper extremity motor function score differs significantly at each time point. The findings also show that there was no statistically significant interaction effect between time and gender [*F*(1.42) = 2.103, *p* > 0.05] or between time and stroke type [*F*(1.31) = 0.364, *p* > 0.05] for UEMF.

### 3.6. Clinical Significance for Motor Function Performance (MFP), Lower Extremity Motor Function (LEMF), and Upper Extremity Motor Function (UEMF) among Stroke Patients with a Very Severe Motor Impairment

Table 5 demonstrates that motor function performance was statistically significant between pre- and post-intervention periods. The average difference in total motor function scores between the pre- and post-intervention time points was 17.82. The effect sizes of the differences were 1.35 (large effect sizes). The minimally important differences in motor function scores ranged between 2.65 and 6.62 when using 0.2 and 0.5 effect sizes for calculation. The calculated mean difference values of the difference between pre- and post-intervention time points were higher than the MID values, demonstrating a clinically relevant result (Table 5).

In addition, another additional descriptive analysis based on clinical categories was performed to reinforce the clinically significant motor recovery results. The interpretation of motor impairment is based on Duncan et al. [23], where an overall FMA score of 0 to 35 indicates very severe motor impairment, 36 to 55 indicates severe impairment, 56 to 79 indicates moderate impairment, and more than 79 indicates mild motor impairment. Overall, all participants had improved from very severe motor impairment to moderate (*n* = 1) or severe impairment (*n* = 6) following 12 weeks of exercise.

**Figure 5:**
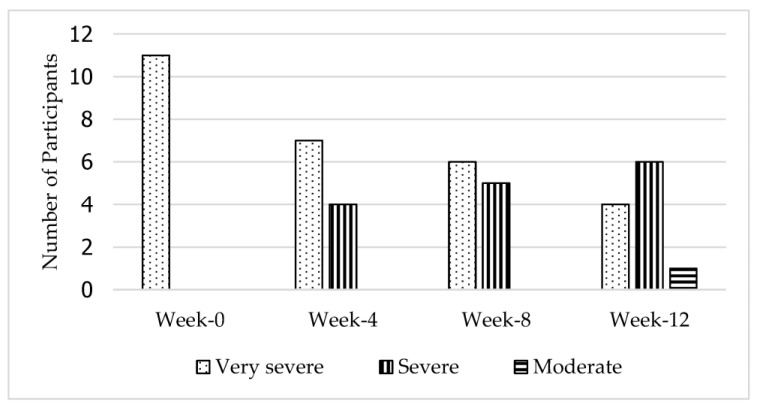
Changes in the severity of motor impairments across the 12-week core exercise intervention.

Figure 5 shows that four subjects had improved to severe motor impairment after 4 weeks of intervention. Only one patient showed an improvement from very severe to severe after 8 weeks of intervention. Finally, after 12 weeks of intervention, one patient was able to progress to moderate impairment, while six patients were classified as having a severe impairment and four patients still had a very severe motor impairment. Therefore, it can be concluded that 12 weeks of core exercise training has a clinically significant effect on motor function recovery in stroke survivors with very severe motor deficits.

Differences in lower extremity motor function were statistically significant between the pre- and post-intervention time points. The average difference in lower extremity motor function was 7.64. The MIDs in lower extremity motor function ranged between 2.43 and 6.08 when using 0.2 and 0.5 as effect sizes for calculation. Because the calculated mean difference value of the difference between pre- and post-intervention was higher than the MIDs value (in addition to large effect sizes), a clinically relevant result was demonstrated (Table 5). Thus, it can be concluded that 12 weeks of core exercise training has a clinically significant effect on improving lower extremity motor function in stroke survivors with very severe motor deficits.

The post-intervention scores for upper extremity motor function were statistically higher than the pre-intervention scores. An average of more than 10 score differences was found. The calculated effect size of the difference was 1.15 (large effect size). The minimal important differences in upper extremity motor function scores ranged between 1.77 and 4.42 when using 0.2 and 0.5 as effect sizes for calculation. The calculated mean difference value of upper extremity motor function between pre- and post-intervention was higher than the mean values (in addition to large effect sizes), demonstrating a clinically relevant result (Table 5). As a result, it can be concluded that 12 weeks of core exercise training has a clinically significant effect on improving upper extremity motor function in stroke survivors with very severe motor deficits.

## 4. Discussion

Stroke affects core performance [24,25,26,27,28], which subsequently causes impairments to core motor control, issues with the patient’s perception of position, and difficulty with coordination and postural adjustment, while also affecting core and extremity functions and impairing balance abilities, gait, and ambulation [29]. The impairments to core musculature not only affected the acute stage but also the chronic stage. Studies have shown that stroke patients still present with mild-to-severe trunk impairment at the chronic stage [30,31]. Another study found that weaker trunk extensor and flexor activations, as well as lower peak torques, have been noted in stroke patients six months after stroke onset when compared to healthy controls [30].

Core deficits following stroke are multidirectional in hemiplegic patients [25,32]. These deficiencies are characterized by core muscle weakness and delayed activation, severe errors in trunk position perception, insufficient control of the center of pressure (CoP), more postural oscillations, difficulty with coordination and postural adjustment, balance impairment, poor core performance and extremity function, and asymmetric trunk kinematics during walking and ambulation [24,25,26,29,33]. The dysfunction in core musculature in hemiparesis stroke leads to functional disability, dysfunction, and dependency [34,35,36]. Stroke patients with severe motor impairments are labelled as “poor candidates” for stroke rehabilitation because of the perceived limitations to their rehabilitation potential and the fact that they do not experience functional improvements comparable to those of stroke survivors in the “middle band” [37]. Thus, this study was carried out to determine whether core exercise can help in improving the motor function of stroke patients with very severe motor impairments.

In this study, overall motor function performance and upper and lower extremity motor function scores increased significantly across the 12-week intervention. The improvement was also significant between all the time point pairs (as shown in Figure 1, Figure 2 and Figure 3). The clinical relevance assessment also shows clinically relevant results with a large effect size for all study variables. This indicates that 12 weeks of core exercise training has a statistically significant and clinically significant effect on motor function performance, upper extremity motor function, and lower extremity motor function in stroke survivors with very severe motor impairments.

Core stability is also an essential core component of coordinated extremity movement, balance, and motor task performance [29]. Increased proximal stability will increase limb mobility [38]. In stroke survivors, poor postural control and core stability lead to poor upper and lower extremity function [39,40,41]. Therefore, we believe increased core stability and core strength following core exercise training contributed to the improvements in motor function performance observed in this study.

In a healthy individual, the muscles in the core contract before those in the upper and lower extremities, which provides dynamic stability by stabilizing the spine, pelvis, and shoulder girdle and building a strong foundation for the powerful movement of the limbs [42]. When these muscles contract synchronously, they enhance movement patterns efficiently. According to Hodges and Richardson [4], who studied the order in which muscles are activated during whole-body movements, muscles such as the transversus abdominis, multifidus, rectus abdominis, and oblique abdominals were consistently recruited before any limb movements. These research findings are consistent with the theory that the development of movement control and stability occurs according to core-to-extremity (proximal-to-distal) progression [3].

The 12 weeks of core exercise training employed in this study involved the use of concurrent training that consisted of both endurance and resistance training. According to Hughes et al. [43], muscle strength, muscle mass, and neural adaptations increase with resistance exercise over time, which begins with neural adaptation that is followed by muscle mass increases and extracellular matrix adaptation to the new stimulus. This means that motor function should continue to improve with longer periods of training. Short et al. [44] showed that 16 weeks of cycling increased mixed muscle protein synthesis by 22% (*p* < 0.05), with age observed to have no effect on training response. However, due to variability in adaptations, the optimal training program for a given individual has not yet been developed [43].

In stroke, lower limb impairment and core control dysfunction have been linked to pelvic instability [45]. The pelvis is a crucial structure that joins the core to the lower extremities, supports the body’s weight, and distributes the load to the lower limbs. The pelvis also serves as a foundation for proximal dynamic stability, allowing for efficient lower limb mobility. Studies have also shown that chronic stroke leads to very weak hip muscles, especially the extensors and adductors [46]. According to Mahmood et al. [33], stroke survivors exhibit decreased core motor control in all planes, with the frontal plane being the most affected. Core motor control refers to the ability of the core muscles to keep the body upright, regulate weight shifts, and conduct selected trunk movements that maintain the base of support during static and dynamic postural modifications [28].

Thus, improvements in abdominal muscle activation, hip muscle strength, and pelvic stability following core exercise training helped to improve lower extremity function in this study. Supported this finding, Olczak [31] demonstrated that active abdominal tension caused an increase in core stability and improved range of movement in the lower extremities, which thus helped patients to achieve a higher level of coordinated lower limb movement.

This study also found that 12 weeks of core exercise training significantly improved upper extremity function in stroke survivors with very severe motor impairments. Previous studies also demonstrated that core control and stability are associated with upper extremity [47,48] and lower extremity function [31] in stroke survivors.

On the other hand, previous studies have also reported that core muscle training showed no significant benefit to upper limb functions [2,5,49], despite some studies having demonstrated that positive correlation exists between core function and upper extremity function [47,50].

This discrepancy in results could be attributable to the shorter training periods (6 weeks) used in earlier research. Studies had shown that upper limb impairment in stroke survivors was greater than in the lower limbs. Even though the degree of weakness between the upper and lower limbs is similar, it was found that the upper limb is weaker than the lower limb [51]. Thus, the six-week training intervention in previous studies may not have been enough to produce a remarkable change in upper extremity function.

In addition, this study also found that the effect of 12 weeks of core exercise training on motor function performance, including upper and lower extremity motor function, is similar for men and women. This finding supports a previous study by Meyer et al. [52], who also found that recovery rate after stroke did not differ between males and females.

Comparison between the ischemic and hemorrhagic group also showed no significant group differences in total motor function, upper motor function, or lower motor function progress. Though the pathophysiology of these types of strokes is different, both ischemic and hemorrhagic stroke patients progress well with core exercise training. This is consistent with previous studies that found no significant difference in functional status between ischemic and hemorrhagic stroke patients after three months of post-acute neurorehabilitation [53,54].

There are a few limitations in this study. First, the sample size was small, and a comparison study could benefit more from a large-scale clinical investigation. Second, pre- and post-change trunk impairment scale components were not assessed in this study. Third, a long-term follow-up was not conducted, thus it is unknown whether core exercise training will lose its benefit over time in patients with hemiplegia. Therefore, additional research must be conducted in the future.

## 5. Conclusions

In conclusion, concurrent core exercise training is effective for improving motor function performance, which comprises upper and lower extremity motor function, in chronic stroke patients with very severe motor impairments. Core exercise training improves core functions that contribute to upper and lower extremity motor function. Exercise targeting the core musculature is important for motor function recovery among chronic stroke patients. This study confirms that functional recovery in stroke patients is highly dependent upon core functions. These findings further emphasize the need to prescribe core muscle exercises progressively according to the physical ability of stroke patients.

Future research will have the potential to evaluate the effect of core muscle exercise on motor function recovery in acute and sub-acute stroke patients. Concurrently, further investigation should be carried out to determine neuroplasticity.

## Figures and Tables

**Table 1:** Core exercise training guidelines.

Mode of Exercise	Pre-Requisite	Activity
Level 1: *Supine exercise* ▪ With or without assistance ▪ 8 exercises	None.	▪ Glute bridge ▪ Holding knee to chest ▪ Knee marches ▪ Hip abduction–adduction with knees bent or straight legs ▪ Hip rotation with knees bent ▪ Hip-knee flexion–extension ▪ Abdominal crunch (with shoulder off the floor) ▪ Lateral trunk rotation ▪ Shoulder flexion (bilateral arm movement) ▪ I’ Y’ T (bilateral arm movement)
Level 2: *Sitting exercise* ▪ With or without a resistance band ▪ With or without assistance ▪ 8 exercises	Static posture, sitting balance, and stability.	▪ Shoulder extension with back hyperextension ▪ Arm row ▪ Chest press ▪ Trunk forward side bend ▪ Lateral trunk flexion ▪ Trunk forward reach ▪ Trunk rotation ▪ Hip abduction–adduction
Level 3: *Core strengthening* ▪ Load (10–15 RM) ▪ With or without assistance ▪ 5 exercises	Dynamic posture, stability, and mobility.	▪ Back extension ▪ Leg press ▪ Chest press ▪ Abdominal crunch ▪ Lat pull-down or low row ▪ Hip rotator
Level 4: *Dynamic core stabilization* ▪ With or without a resistance band ▪ With or without support ▪ 5 exercises	Joint stability, dynamic balance, and mobility.	**Bend and lift** Level 1: squat Level 2: overhead squat **Push** Level 1: standing chest press (feet shoulder-width apart) Level 2: dumbbell overhead press **Pull** Level 1: standing row Level 2: standing staggered stance one arm row **Single leg** Level 1: standing leg balance (horizontal abduction, frontal reach, diagonal reach) Level 2: half kneeling rising lunges **Rotation** Level 1: thoracic rotation Level 2: body swing

**Table 2:** Characteristics of patients.

**Variable**	**Category**	**Total** **(*n* = 11)**	**Gender**				
			**Male** **(*n* = 6)**	**Female** **(*n* = 5)**	**df**	**t**	***p***
Time since onset, *mean ± s.d., months*		12 ± 7	13.0 ± 8.76	10.2 ± 3.49	6.78	0.72	>0.05
Age, *mean ± s.d., years*		41 ± 10	45.3 ± 5.72	36.2 ± 11.61	5.60	1.61	>0.05
					**df**	***X*** ^2^	***p***
Age, *n (%)*	20–29 years	2 (18.2%)	-	2 (40%)	3	4.14	>0.05
	30–39 years	2 (18.2%)	1 (16.7%)	1 (20%)			
	40–49 years	5 (45.5%)	3 (50%)	2 (40%)			
	50–59 years	2 (18.2%)	2 (33.3%)	-			
Stroke type, *n (%)*	Ischemic	5 (45.5%)	2 (33.3%)	3 (60%)	1	0.78	>0.05
	Hemorrhagic	6 (54.5%)	4 (66.7%)	2 (40%)			
Hemiparesis side, *n (%)*	Left	5 (45.5%)	3 (50%)	2 (40%)	1	0.11	>0.05
	Right	6 (54.5%)	3 (50%)	3 (60%)			

**Table 3:** Demographic profile for age categories.

Variable		Age Category			
		20–29 (*n* = 2)	30–39 (*n* = 2)	40–49 (*n* = 5)	50–59 (*n* = 2)
Time since onset, *mean ± s.d., months*		11 ± 4	8 ± 1	12 ± 8	14 ± 7
Age, *mean ± s.d., years*		24 ± 0	38 ± 2	46 ± 2	51 ± 1
Gender, *n (%)*	Male	-	1 (50%)	3 (60%)	2 (100%)
	Female	2 (100%)	1 (50%)	2 (40%)	-
Stroke type, *n (%)*	Ischemic	2 (100%)	1 (50%)	-	2 (100%)
	Hemorrhagic	-	1 (50%)	5 (100%)	-
Hemiparesis side, *n (%)*	Left	-	-	4 (80%)	1 (50%)
	Right	2 (100%)	2 (100%)	1 (20%)	1 (50%)

**Table 4:** Summary of repeated measures ANOVA analysis to determine the main effect of time (t), the interaction effect of time and gender, and the interaction effect of time and stroke type.

	Wk0	Wk4	Wk8	Wk12	Repeated Measures ANOVA								
					Main Effect (Time)			Interaction Effect (Time × Gender)			Interaction Effect (Time × Stroke Type)		
					df	F	P	df	F	P	df	F	P
MFP (mean ± s.d.)	21.09 ± 12.01	28.27 ± 13.41	32.45 ± 13.47	38.91 ± 14.37	1.62	57.164	<0.01	1.62	0.726	>0.05	1.46	0.398	>0.05
LEMF (mean ± s.d.)	7.73 ± 4.78	11.09 ± 5.43	12.91 ± 5.38	15.36 ± 5.39	1.68	32.509	<0.01	1.68	0.501	>0.05	1.75	1.068	>0.05
UEMF (mean ± s.d.)	13.36 ± 7.51	17.18 ± 8.47	19.55 ± 9.04	23.55 ± 9.970	1.42	28.282	<0.01	1.42	2.103	>0.05	1.31	0.364	>0.05

**Table 5:** Summary of clinical relevance assessment of functional outcomes in stroke survivors with very severe motor impairments.

Outcome	Mean Diff.	CI for the Mean Difference		Pooled SD	Effect Size (ES) or Standardized Mean Difference	Interpretation ES	MID (0.2) = 0.2 × Pooled SD	MID (0.5) = 0.5 × Pooled SD	Final Decision on Clinical Relevance
		Lower	Upper						
Post MFP vs. Pre MFP	17.82	13.22	22.41	13.24	1.35	LES	2.65	6.62	CR
Post LEMF vs. Pre LEMF	7.64	5.23	10.05	12.16	1.50	LES	2.43	6.08	CR
Post UEMF vs. Pre UEMF	10.18	6.04	14.32	8.83	1.15	LES	1.77	4.42	CR

MFP: motor function performance; LEMF: lower extremity motor function; UEMF: upper extremity motor function; NCR: not clinically relevant; PCR: potentially clinically relevant; CR: clinically relevant; SES: small effect size; MES: moderate effect size; LES: large effect size.

## Data Availability

The data presented in this study are available on request from the corresponding author. The data are not publicly available due to confidentiality issues.

## Abbreviations

FMA	Fugl-Meyer Assessment
FMA-LE	Fugl-Meyer Assessment Lower Extremity
FMA-UE	Fugl-Meyer Assessment Upper Extremity
MMSE	Mini-Mental State Examination
ES	effect size
MIDs	minimal important differences
CR	clinically relevant
PCR	potentially clinically relevant
NCR	not clinically relevant
MFP	motor function performance
LEMF	lower extremity motor function
UEMF	upper extremity motor function
SES	small effect size
MES	moderate effect size
LES	large effect size
